# Neurological Complications Associated With SARS-CoV-2 Infection: A Single-Centre Experience

**DOI:** 10.7759/cureus.32655

**Published:** 2022-12-18

**Authors:** Mariana Martins, Ana Pereira, André Teixeira, Diana Lima, Nuno Lopes, Marta Amaral-Silva, Isabel Seixo, Ana Catarina Miguéns

**Affiliations:** 1 Physical Medicine and Rehabilitation, Centro Hospitalar Universitário de Lisboa Central, Lisbon, PRT

**Keywords:** coronavirus disease 2019, sars-cov-2, covid-19, rehabilitation, critical ill, prone position, covid-19 patients, neurological complications

## Abstract

Background: The clinical presentation of severe acute respiratory syndrome coronavirus 2 (SARS-CoV-2) infection can range from mild or moderate disease (80% of the cases) to severe disease (15%) requiring oxygen support, and critical disease (5%), associated with acute respiratory distress syndrome and admission to the intensive care unit (ICU). In critically ill patients, prone positioning can be used to optimize oxygenation. Although there is a favourable response to this strategy, being a life-saving measure, additional associated complications may appear, including compressive neuropathies. Despite respiratory affection being more common, SARS-CoV-2 infection can also attack other systems and can, under certain conditions, affect the central or peripheral nervous system. It has been described that neurological manifestations can result from the neuroinvasive properties of the SARS-CoV-2 or as an indirect consequence of multiorgan dysfunction.

Aims: We intend to report the patients who presented with neurological complications associated with coronavirus disease 2019 (COVID-19) and/or complications of its treatment, followed in our physical and rehabilitation medicine (PRM) service.

Materials and methods: A retrospective analysis of patients admitted to the PRM ward with outpatient consultation in the context of post-COVID-19 status between April 2020 and November 2021 (the period of the highest prevalence of infection) was carried out. Patients with neurological complications after SARS-CoV-2 infection and consequently a decline in previous functionality were identified.

Results: Thirteen patients (23.6%) admitted to the PRM ward had peripheral neurological complications, documented by electroneuromyography, including Guillain-Barré syndrome, sensory-motor polyneuropathy, peroneal nerve injury, femoral nerve injury, and lumbar plexus injury. The neurological complications of the patients followed in a post-COVID-19 consultation were also evaluated. Eight patients (20%) reported neurological sequelae. Five patients presented peripheral nerve damage (peroneal, accessory, ulnar, and recurrent laryngeal) of undefined aetiology, diagnosed after the acute phase of hospitalization. Two patients had COVID-19 infection followed by ischemic stroke (vertebrobasilar and middle cerebral artery), requiring hospitalization in the acute phase. One patient had COVID-19 infection followed by longitudinal myelitis, with positive anti-myelin oligodendrocyte glycoprotein (MOG). All patients required follow-up by the rehabilitation team with partial recovery of deficits.

Conclusions: All patients admitted to the PRM ward with neurological manifestations had critical disease and symptoms compatible with peripheral nervous system involvement. Patients admitted to the PRM consultation had different levels of viral disease severity and had sequelae related to peripheral and central nervous system disorders. Identifying the aetiology of these injuries is essential for us to act on their prevention, particularly with regard to indirect complications, such as compressive neuropathies. It will be necessary to maintain the follow-up of these patients to understand the evolution of the neurological consequences associated with COVID-19.

## Introduction

The first cases of coronavirus disease 2019 (COVID-19) were reported in China in December 2019. On March 11 2020, the World Health Organization characterized COVID-19 as a pandemic disease [1]. SARS-CoV-2, a strain of coronavirus (CoV), causes the clinical syndrome COVID-19, whose pulmonary manifestations have been well recorded [2]. Less frequent symptoms have been described, such as dermatological manifestations, gastrointestinal symptoms, cardiovascular events, and neurological complications.

First data on COVID-19 suggested neurological involvement in a variable percentage of cases, with particular expression in more severe patients who presented a systemic response to the virus. A first retrospective series on 214 hospitalized patients reported neurological symptoms in about 36% of them [1]. Neurological symptoms of COVID-19 can be grouped into the central nervous system (CNS) and peripheral nervous system (PNS) affection and can cause both acute and long-term neurological complications [3]. The heterogeneity of clinical, pathological, and radiological presentations of neurological manifestations associated with COVID-19 suggests that different pathogenic pathways could be involved [3,4]. It has been mentioned in the literature that neurological involvement in COVID-19 can be correlated with a variety of hypotheses such as neurological manifestations of viral infection, postinfectious neurological complications, infection in patients with neurological comorbidity and worsening of its previous state, and neurological side effects associated with COVID-19 treatments [3,4]. Damage to the nervous system with viral neuroinvasion may be achieved by some routes, including via olfactory nerve, transsynaptic transfer across infected neurons, leukocyte migration across the blood-brain barrier, or infection of the vascular endothelium [4-6].

The most common neurological symptoms of COVID-19 are dizziness, headache, anosmia, and ageusia. More severe neurological manifestations include stroke, impairment of consciousness, seizures, and encephalopathy, luckily less often [7]. Acute cerebrovascular diseases were reported in 0.7-5.8% of hospitalized COVID-19 patients, with a predominance of acute ischemic stroke, followed by intracranial haemorrhage and cerebral venous thrombosis [1]. The causal relationship between SARS-CoV-2 and stroke has been studied and a recent study showed that the risk of stroke was significantly higher with SARS-CoV-2 infection than with influenza infection, after adjustment for vascular risk factors [1]. In the literature evaluated, strokes in COVID-19 patients were significantly more severe and with poorer outcomes, which may be associated with these injury mechanisms beyond conventional cardiovascular risk factors [8]. Beyond CNS complications detected, some studies have described up to 8% of patients with PNS-related complications [9]. Postinfectious immune-mediated mechanisms such as acute disseminated encephalomyelitis (ADEM), acute transverse myelitis, and Guillain-Barré syndrome (GBS) and its variants have been reported [1,2,10]. Additionally, about 10% of hospitalized patients need assistance in intensive care wards, highlighting the need to assess the "critical illness neuro-myopathy", known to delay weaning from ventilation, with a very marked functional impact, culminating in longer hospital stays, associated with greater complications and representing a significant burden on the healthcare delivery system [1-3,11,12].

A great effort was required from worldwide health systems with the SARS-CoV-2 pandemic. In Lisbon, the Hospital Curry Cabral was a key player in the fight against the virus, having suspended normal, unscheduled hospital activity, to respond to the growing number of cases of infection. With the growing number of patients recovering from SARS-CoV-2 infection, the rehabilitation issue has become a crucial one. Early evidence for the need for rehabilitation, including neurological aspects, in clinically recovered patients, has been demonstrated. Although valid information about neurological complications and their impact is still being formed, we intend to report a case series of patients with neurological complications associated with SARS-CoV-2, associated with the disease or complications of its treatment, followed in our physical and rehabilitation medicine (PRM) service.

## Materials and methods

Patients with SARS-CoV-2 infection admitted to Hospital Curry Cabral were hospitalized according to their clinical status, either in the intensive care unit, internal medicine unit, or infectious diseases unit. An interdisciplinary rehabilitation program designed to cross all phases of the patient’s hospitalization was established to optimize neuromotor and respiratory conditions. As Curry Cabral Hospital includes a PRM inpatient care service, patients with severe functional impairment due to prolonged hospitalization were transferred from acute care services into the rehabilitation unit. Admission to the rehabilitation unit was proposed to patients once they met the clinical cure criteria for coronavirus (as they had to safely move into the different rehabilitation sections) and when they were sufficiently stable to actively participate in therapy in all cases that the patient presented severe impairment in mobility, affecting independence and functionality, severe impairment in performing basic activities of daily living, and other clinical conditions with significant impact in functional status.

Inpatient rehabilitation aims are to improve the consequences of immobilization and facilitate home discharge. Unlike other patients admitted to these units, COVID-19 patients were expected not to be able to tolerate intensive therapies due to severe respiratory impairment. During this period, patients were included in an intensive rehabilitation program, including, as needed, physiotherapy, occupational therapy, speech therapy, and rehabilitation nursing assistance.

After discharge, all patients treated by the PRM team during hospitalization were telemonitored to trace possible consequences of the disease, mainly in the domain of the cardiorespiratory and motor functions. Patients who received guidance about self-rehabilitation programs to perform at home were asked about compliance and adherence to treatment and were advised of maximum adherence. Rehabilitation needs accessed at this point were given proper counselling. Taking into account the great evolution and bearing in mind the need to reassess patients with COVID-19 sequelae, a specialist consultation was created to provide an appropriate response. In this consultation, they were referred in the following circumstances: reassessment of patients after discharge from the physical medicine and rehabilitation service, who may be referred to a rehabilitation program on an outpatient basis or on an outpatient basis in the area of ​​residence, if indicated; reassessment of patients observed by the rehabilitation team as impatient in internal medicine or infectious diseases, having started a rehabilitation program in the ward, and with the need to continue this rehabilitation care; referral of patients with COVID-19 sequelae by colleagues from the acute wards, whether they have respiratory symptoms, depressive symptoms, symptoms of loss of physical conditioning, immobility syndrome sequelae, musculoskeletal complaints or neurological sequelae associated with viral infection or prolonged hospitalization itself.

A retrospective analysis of patients admitted to the PRM ward with outpatient consultation in the context of post-COVID-19 status between April 2020 and November 2021 (the period of the highest prevalence of infection) was carried out.

Central or peripheral neurological signs were searched. Patients with neurological complications after SARS-CoV-2 infection and consequently a decline in previous functionality were identified. Common light symptoms such as headache, dizziness, and changes in taste or smell were not included. The following data were also recorded: demographic data, comorbidities, characterization of the COVID-19 severity during hospitalization (mild/moderate, severe, and critical), length of stay in the ICU and invasive mechanisms if indicated (average days), need for prone ventilation or extracorporeal membrane oxygenation, need for hospitalization in PRM ward and functional evolution with the application of evaluation scales at admission and discharge, and follow-up and consultations.

Data from the selected patients were retrospectively entered into a Microsoft Excel sheet (Microsoft Corporation, Redmond, WA). Categorical data are presented as numbers and percentages. A descriptive statistical methodology was applied to the data. Patients were not directly studied, and analysis was based on retrospective data collection without the identification of patients.

## Results

In the period analysed, 55 patients were admitted to the PRM ward for sequelae of severe SARS-CoV-2 infection, with neuromotor sequelae and loss of functionality. Of these, 13 patients (69.2% male and mean age of 57.4 years) had peripheral neurological complications, documented by electroneuromyography, including Guillain-Barré syndrome (n = 2, 15.4%), sensory-motor polyneuropathy (n = 3, 23.1%), peroneal nerve injury (n = 6, 46.2%), femoral nerve injury (n = 1, 7.7%), and lumbar plexus injury (n = 1, 7.7%) (Table 1).

**Table 1:** Reports of EMG-NCS EMG-NCS: electromyography and nerve conduction studies.

Patient's age and gender	Reports of EMG-NCS
71, male	Left femoral nerve injury, subacute, located above the emergence of the branch that innervates the psoas iliac muscle, axon, and with severe severity.
62, female	Sensory-motor polyneuropathy, predominantly axonal and in the lower limbs and distal segments.
45, male	Active denervation in all the muscles studied, more evident in the proximal muscles - neuromyopathy in intensive care with predominant muscle involvement is considered possible, the severity of which cannot be established due to lack of cooperation from the patient. Additionally, the discrepancy between neurography findings of the posterior tibial and left peroneal nerves suggests an additional neuropathy of the latter nerve.
39, male	Examination compatible with the diagnosis of axonal polyneuropathy, with greater involvement of the lower limbs and distal segments.
67, male	Motor-predominant demyelinating sensory-motor polyneuropathy, supporting the hypothesis of Guillain-Barré syndrome (form acute inflammatory demyelinating polyradiculopathy (AIDP)).
66, female	Motor-predominant demyelinating sensory-motor polyneuropathy, supporting the hypothesis of Guillain-Barré syndrome (form AIDP).
57, female	Bilateral axonal damage of common peroneal nerves, subacute, located above the emergence that innervates the lateral peroneal muscles, severe left and moderate right severity.
68, female	Bilateral axonal damage to the common peroneal nerves, subacute, located above the emergence that innervates the lateral peroneal muscles, severe left and moderate right severity.
63, male	Partial lesion of the left lumbar plexus, subacute, predominantly axonal.
56, male	The exam is compatible with the diagnosis of an axonal type of injury to the right common peroneal nerve, of severe severity (no signs of reinnervation in all muscles studied).
55, male	The examination is compatible with the diagnosis of bilateral common peroneal nerve injury, subacute axonal type and severe severity.
55, male	The examination is compatible with the diagnosis of non-necrotizing myopathy with greater involvement of the proximal muscles. Additionally, the discrepancy between neurographic and electromyographic findings of the dependent muscles of the posterior tibial and peroneal nerves suggests an additional neuropathy of the peroneal nerves.
42, male	The examination is compatible with the diagnosis of bilateral common peroneal nerve injury, subacute axonal type and severe severity.

All the patients had a critical illness with acute respiratory distress syndrome requiring hospitalization in the ICU and invasive mechanical ventilation (IMV) (mean: 33.7 days). Of the patients, 76.9% (n = 10) required prone positioning, on average 2.4 periods with a minimum duration of 16 hours, and 30.8% required extracorporeal membrane oxygenation (ECMO).

All patients who required ECMO were previously positioned in the prone position, without success. All patients with peroneal nerve injury, either unilateral or bilateral, were positioned in the prone position. Table 2 shows the correlation between these data.

**Table 2:** Foot drop statistic data ICU: intensive care unit; ECMO: extracorporeal membrane oxygenation; EMG-NCS: electromyography and nerve conduction studies.

Age	Gender	ICU, number of days	Prone ventilation, number of periods	ECMO	Medical comorbidities	Foot drop	Side	EMG-NCS
57	Female	Yes, 42	Yes, 2	No	Obesity	Yes	Left	Yes
68	Female	Yes, 27	Yes, 2	No	Hypertension, obesity, dyslipidaemia, depressive syndrome	Yes	Bilateral	Yes
56	Male	Yes, 18	Yes, 1	Yes	Hypertension, obesity, depressive syndrome	Yes	Left	Yes
55	Male	Yes, 48	Yes, 2	Yes	Hypertension, dyslipidaemia	Yes	Bilateral	Yes
55	Male	Yes, 52	Yes, 2	No	Hypertension, kidney transplant	Yes	Bilateral	Yes
42	Male	Yes, 94	Yes, 10	Yes		Yes	Bilateral	Yes

Regarding comorbidities, 76.9% of patients had cardiovascular risk factors, including arterial hypertension, dyslipidaemia, obesity, and smoking. None of the patients had previous respiratory or neurological pathology.

**Figure 1:**
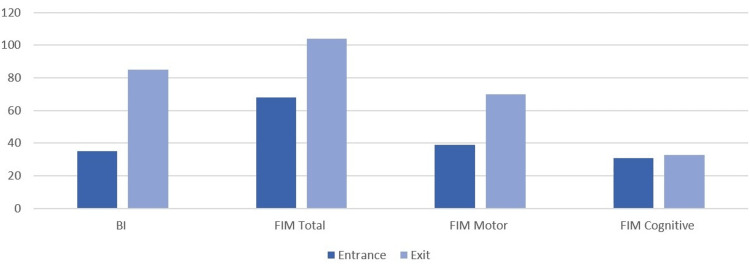
Evolution in functionality scales during the internment in the physical medicine and rehabilitation ward FIM: Functional Independence Measure; BI: Barthel Index.

Patients were transferred to the PRM ward, with an average value of 34.5 in the Barthel Index (BI) and 67.6 in the Functional Independence Measure (FIM) at admission. The average number of days spent in the PRM service was 45.5 days. At discharge, they had a mean value of 85.4 in the BI (evaluation of 50.9 points) and 104 in the FIM (evaluation of 36.4 points) (Figure 1).

After discharge, most (61.5%) patients continued rehabilitation outpatient treatment. Of the patients, 46.2% used support products to improve their gait pattern, including knee-stabilizing orthosis with lateral lock and ankle-stabilizing orthosis (foot-up).

During the analysed period, 40 patients still needed to be followed up at the PRM consultation due to loss of functionality. The neurological complications of these patients followed in a post-COVID-19 consultation were also evaluated, completing 20% ​​of the sample (n = 8). All patients with these neurological alterations were previously evaluated by the rehabilitation team in an acute illness inpatient regime. However, unlike patients in need of an intensive rehabilitation program on an inpatient basis, not all of them had a critical illness requiring hospitalization in intensive care. And in fact, only two patients required prone ventilation and only for a period. This leads us to believe that the severity of the disease in these patients will, in general, be less marked.

Five patients presented peripheral nerve damage (peroneal, accessory, ulnar, and recurrent laryngeal) of undefined aetiology, diagnosed after the acute phase of hospitalization. Two patients had COVID-19 infection followed by ischemic stroke (vertebrobasilar and middle cerebral artery) requiring hospitalization in the acute phase. One patient had COVID-19 infection followed by longitudinal myelitis, with positive anti-myelin oligodendrocyte glycoprotein (MOG). All patients required follow-up by the rehabilitation team with partial recovery of deficits. The evaluated data are reported in Table 3.

**Table 3:** Statistical summary of patients followed in physical medicine and rehabilitation consultation with neurological manifestations ICU: intensive care unit; ECMO: extracorporeal membrane oxygenation; EMG-NCS: electromyography and nerve conduction studies.

Age	Gender	COVID-19 hospitalization	ICU	Prone ventilation	ECMO	Medical comorbidities	Neurologic sequelae	EMG-NCS
65	Male	Yes	Yes, 7	No	No		Peroneal nerve injury	Yes
40	Male	Yes	Yes, 27	Yes, 5	No		Accessory nerve injury	Yes
55	Male	Yes	Yes, 7	Yes, 1	No	Hypertension, dyslipidaemia	Ulnar nerve injury	Yes
37	Male	Yes	No	No	No	Obesity	MOG antibody-associated disease	No
64	Female	Yes	Yes, 5	No	No	Hypertension	Ulnar nerve injury	Yes
67	Female	Yes	No	No	No	Hypertension	Stroke	No
41	Female	Yes	No	No	No		Stroke	No
28	Male	Yes	Yes, 34	Yes, 1	No		Recurrent laryngeal nerve	No

## Discussion

Recognition of SARS-CoV-2-related neurological disease can be challenging, mainly because of the variable evolution of the symptomatology, prognosis, and current lack of exact knowledge of the pathophysiology [1].

However, the proportion of SARS-CoV-2 infections leading to neurological disease probably will remain small, compared to other organ systems affection, especially the respiratory system. Still, despite corresponding to a smaller percentage of patients, these might be left with severe neurological sequelae with prolonged disability [2]. With the increase in affected patients, the proportion of neurological patients and their associated health burden and economic costs might be large [10,13].

The heterogeneity of clinical neurological manifestations associated with SARS-CoV-2 infections suggests that different pathogenic pathways are involved [14-16]. The most severe form of COVID-19 results in an exacerbated systemic inflammatory response, with the release of pro-inflammatory cytokines and a hypercoagulable state [17,18]. The predisposition to thrombotic complications is associated with an unfavourable prognosis, and therefore, pharmacological thromboprophylaxis is recommended for all patients with COVID-19 admitted to the hospital, with severe/critical illness, in the absence of absolute contraindications [19].

In our study, we want to enhance the neuromotor consequences associated with complications with anticoagulant therapy, which include either spontaneous or traumatic haematoma, which culminated in peripheral nerve injury. We included two patients with a compressive peripheral nerve injury in the context of complicated anticoagulant therapy, femoral nerve injury from spontaneous haematoma, and lumbar plexus injury from a fall with lower limb trauma and compressive haematoma formation. In this context, we would like to warn about the close surveillance of signs and symptoms in critically ill patients, under anticoagulant therapy, which could lead to threatening retroperitoneal haemorrhages, as well as the prevention of falls in anticoagulated patients, especially in the context of muscle weakness associated with critical immobilization syndrome [19].

Regarding the sequelae associated with immobilization syndrome and permanency in intensive care, we already have acknowledged the importance of preventive measures for neuromotor sequelae in this group of patients, namely, positioning measures, to prevent neurological compressive syndromes and early mobilization measures in critical illness [15]. Therefore, the aetiology of patients with neuromotor sequelae associated with severe SARS-CoV2 infection, and associated with indirect sequelae from the treatment of critical illness, is not considered to be different from what is described in the literature in other pathologies [14,16,18].

In our study, we were able to define several iatrogenic complications, associated with lifesaving measures, which should be valued for an early diagnosis and a correct approach as soon as there is clinical stabilization. In our statistics, we consider that we have probable compressive neuropathies associated with ICU positions and lifesaving measures, including peroneal and cubital neuropathies; all patients with this type of diagnosis, confirmed by electroneuromyography, remained in the ICU for several days and repositioning with the prone position was necessary. Also, we cannot underestimate recurrent laryngeal nerve palsy after orotracheal intubation with a marked impact on voice quality and swallowing safety or less common compressive neuropathies, such as accessory nerve disorders.

Study limitations

The following are the limitations of the study: a relatively small sample of patients; variability in statistical data with very different patient ages, comorbidities, and layouts, which makes it difficult to predict correlations between them; patients referred for the PRM consultation are potentially the most serious and therefore there may be a selection bias, which it was not possible to control. Despite these study limitations, it was important for us to understand the type of the most seriously ill patients that we had during the peak phase of the pandemic, and to report that most patients with severe or critical illness had associated neurological complications that hampered the clinical and functional evolution.

## Conclusions

All patients admitted to the PRM inpatient department with neurological manifestations have symptoms compatible with PNS involvement. In some cases, it could be a direct consequence of the viral infection being associated with different pathological pathways such as the neuroinvasive capacity of the coronavirus or the result of systemic inflammation that may culminate in multiorgan dysfunction. In other cases, it could be an indirect consequence, as a result of complications related to the instituted therapy and prolonged periods of immobilization, mainly in the critical phase. Identifying the aetiology of these injuries is essential for us to act on their prevention, particularly with regard to indirect complications, such as compressive neuropathies, resulting probably from the positioning of patients. All patients admitted to the PRM ward had a critical illness, requiring intensive care and with a very marked clinical and functional affection, conditioning the need for an intensive rehabilitation program. Unlike these patients who had severe limitations both at a neuromotor and respiratory level, the patients followed in PRM consultation (outpatients) had a more variable severity of COVID-19, with the affection of both the CNS and/or the PNS, becoming more difficult to correlate between disease severity and neurological manifestations, whether associated with direct or indirect consequences.

During the pandemic, rehabilitation attendance was important in monitoring patients from the critical period until discharge and preventing complications associated with a prolonged hospital stay. Early rehabilitation and mobilization in critical patients are essential to prevent sequelae. It will be necessary to maintain the follow-up of these patients to understand the evolution of the neurological consequences associated with COVID-19.
